# *N*,*N*′-Bis(pyridin-2-yl)benzene-1,4-diamine–naphthalene (2/1)

**DOI:** 10.1107/S1600536811047519

**Published:** 2011-11-12

**Authors:** Barbara Wicher, Maria Gdaniec

**Affiliations:** a Faculty of Chemistry, Adam Mickiewicz University, 60-780 Poznań, Poland

## Abstract

The asymmetric unit of the title compound, C_10_H_8_·2C_16_H_14_N_4_, consists of one mol­ecule of *N*,*N*′-bis­(pyridin-2-yl)benzene-1,4-diamine (PDAB) and one half of the centrosymmetric naphthalene mol­ecule. The PDAB mol­ecule adopts a non-planar conformation with an *E* configuration at the two partially double *exo* C N bonds of the 2-pyridyl­amine units. In the crystal, N—H⋯N hydrogen bonds between the PDAB mol­ecules generate a cyclic *R*_2_^2^(8) motif, leading to the formation of PDAB tapes extending along [100]. The tapes are arranged into (010) layers and the naphthalene mol­ecules are enclosed in cavities formed between the PDAB layers.

## Related literature

For the structures of polymorphic forms of *N*,*N*′-di(pyridin-2-yl)benzene-1,4-diamine, see: Bensemann *et al.* (2002 ▶); Wicher & Gdaniec (2011*a* ▶). For the structures of *N*,*N*′-di(pyridin-2-yl)benzene-1,4-diamine co-crystals with phenazine and quin­oxaline, see: Gdaniec *et al.* (2005 ▶); Wicher & Gdaniec (2011*b* ▶).
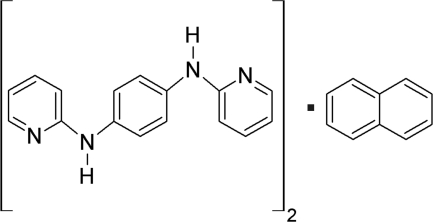


## Experimental

### 

#### Crystal data

C_10_H_8_·2C_16_H_14_N_4_
*M* *_r_* = 652.78
Monoclinic, 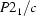

*a* = 9.2224 (1) Å
*b* = 22.8371 (2) Å
*c* = 8.8760 (1) Å
β = 117.936 (2)°
*V* = 1651.56 (3) Å^3^
*Z* = 2
Cu *K*α radiation
μ = 0.63 mm^−1^
*T* = 130 K
0.20 × 0.15 × 0.05 mm

#### Data collection

Oxford Diffraction SuperNova diffractometer
Absorption correction: multi-scan (*CrysAlis PRO*; Agilent, 2010 ▶) *T*_min_ = 0.931, *T*_max_ = 1.000
9878 measured reflections
3020 independent reflections
2671 reflections with *I* > 2/s(*I*)
*R*_int_ = 0.019

#### Refinement

*R*[*F* ^2^ > 2σ(*F* ^2^)] = 0.033
*wR*(*F* ^2^) = 0.091
*S* = 1.05
3020 reflections
226 parameters
H-atom parameters constrained
Δρ_max_ = 0.17 e Å^−3^
Δρ_min_ = −0.19 e Å^−3^

### 

Data collection: *CrysAlis PRO* (Agilent, 2010 ▶); cell refinement: *CrysAlis PRO*; data reduction: *CrysAlis PRO*; program(s) used to solve structure: *SIR2004* (Burla *et al.*, 2005 ▶); program(s) used to refine structure: *SHELXL97* (Sheldrick, 2008 ▶); molecular graphics: *ORTEP-3 for Windows* (Farrugia, 1997 ▶) and *Mercury* (Macrae *et al.*, 2006 ▶); software used to prepare material for publication: *SHELXL97*.

## Supplementary Material

Crystal structure: contains datablock(s) global, I. DOI: 10.1107/S1600536811047519/rz2666sup1.cif

Structure factors: contains datablock(s) I. DOI: 10.1107/S1600536811047519/rz2666Isup2.hkl

Supplementary material file. DOI: 10.1107/S1600536811047519/rz2666Isup3.cml

Additional supplementary materials:  crystallographic information; 3D view; checkCIF report

## Figures and Tables

**Table 1:** Hydrogen-bond geometry (Å, °)

*D*—H⋯*A*	*D*—H	H⋯*A*	*D*⋯*A*	*D*—H⋯*A*
N7—H7*N*⋯N16^i^	0.90	2.11	3.0027 (13)	175
N14—H14*N*⋯N2^ii^	0.90	2.13	3.0305 (13)	174

Symmetry codes: (i) 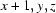; (ii) 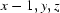.

**Fig. 1.**
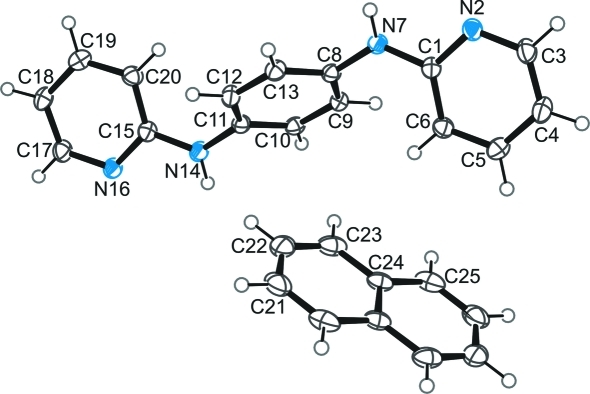
: Asymmetric unit of the title compound with displacement ellipsoids shown at the 50% probability level. The unlabelled atoms are related to the labelled ones by the symmetry operation 1 - x, -y, 1 - z.

**Fig. 2.**
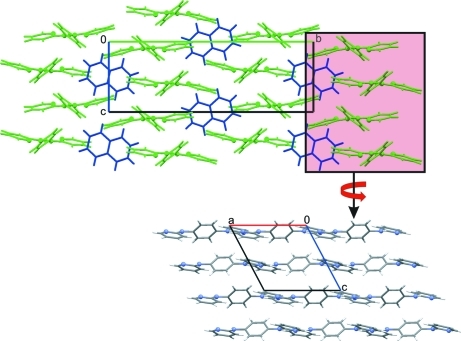
: Crystal packing in the title compound viewed along the a axis and the view of the (0 1 0) layer formed by the hydrogen-bonded PDAB tapes.

## supplementary crystallographic information

### Comment

*N*,*N*'-Di(pyridin-2-yl)benzene-1,4-diamine (PDAB) is a very versatile supramolecular reagent (Bensemann *et al.*, 2002; Gdaniec *et al.*, 2005). Recently we have shown that this compound can form 2:1 cocrystals with an aromatic heterobase quinoxaline (Wicher & Gdaniec, 2011*b*). In these cocrystals PDAB molecules were organized into hydrogen-bonded tapes with N—H···N hydrogen bonds occurring between self-complementary 2-pyridylamine groups. The quinoxaline molecule enclosed in a centrosymmetric cage, due to disorder, simulated very well the naphthalene molecule. We have concluded that PDAB when cocrystalized with naphthalene should form isostructural crystals. Indeed, cocrystals of PDAB with naphthalene with the 2:1 component ratio were obtained, however, to our surprise, they were not isostructural with their quinoxaline analogue.

The title complex is shown in Fig. 1. The PDAB molecule is nonplanar and adopts an *E*,*E* form that promotes the formation of a cyclic *R*^2^_2_(8) motif *via* N—H···N hydrogen bond between the self-complementary 2-pyridylamine units (Table 1). These cyclic motifs assemble PDAB molecules into tapes extending along [1 0 0]. The structure of the tape is very similar to that formed in the cocrystal with quinoxaline, however the arrangement of the tapes is different. In the cocrystal with quinoxaline the tapes were arranged into pairs *via* aromatic π···π stacking interactions, whereas in the title cocrystal they are arranged into (0 1 0) layers with a short C–H···C contact [H12···C9^i^ 2.79 Å, <C12–H12···C9^i^ 171°; symmetry code (i): *x*, 0.5 - *y*, 1/2 + *z*] occurring between the PDAB molecules. The naphthalene molecules are enclosed in cages formed between adjacent (0 1 0) layers where they also form short C—H···C contacts with PDAB (H17···C21^i^ 2.78 Å, <C17–H17···C21^i^137°; H23···C10 2.81 Å, <C23–H23···C10 147°; symmetry code (i): *x* - 1, 0.5 - *y*, *z* - 1/2).

Looking for the reason for this structural alteration we have noticed that in the cocrystal with quinoxaline the guest molecule enclosed in a cavity forms with PDAB one short H···H contact of 2.09 Å. In the case of naphthalene there would be two such contacts, most probably repulsive in their nature, and therefore sufficiently destabilizing the crystal structure for it being rebuild.

### Experimental

*N*,*N*'-Di(pyridin-2-yl)benzene-1,4-diamine (0.03 g, 0.11 mmol) and naphthalene (0.014 g, 0.11 mmol) were dissolved in 0.75 ml of butanone and placed in a closed plastic vial. After a few days, when butanone solution almost completely evaporated, colourless, plate-shaped crystals were obtained.

### Refinement

H atoms of the N—H groups were located in difference electron density maps. N—H bond lengths were standardized to 0.90 Å and *U*_iso_(H) values were constrained to 1.2*U*_eq_(N). All other H atoms were initially identified in difference electron density maps but were placed at calculated positions, with C—H = 0.95 Å, and were refined as riding on their carrier atoms, with *U*_iso_(H) = 1.2*U*_eq_(C).

### Crystal data

**Table:**

C_10_H_8_·2C_16_H_14_N_4_	*F*(000) = 688
*M**_r_* = 652.78	*D*_x_ = 1.313 Mg m^−^^3^
Monoclinic, *P*2_1_/*c*	Cu *K*α radiation, λ = 1.54184 Å
Hall symbol: -P 2ybc	Cell parameters from 7079 reflections
*a* = 9.2224 (1) Å	θ = 3.9–75.7°
*b* = 22.8371 (2) Å	µ = 0.63 mm^−^^1^
*c* = 8.8760 (1) Å	*T* = 130 K
β = 117.936 (2)°	Plate, colourless
*V* = 1651.56 (3) Å^3^	0.20 × 0.15 × 0.05 mm
*Z* = 2	

### Data collection

**Table:**

Oxford Diffraction SuperNova diffractometer	3020 independent reflections
Radiation source: Nova Cu X-ray Source	2671 reflections with *I* > 2/s(*I*)
mirror	*R*_int_ = 0.019
ω scans	θ_max_ = 68.2°, θ_min_ = 6.7°
Absorption correction: multi-scan (*CrysAlis PRO*; Agilent, 2010)	*h* = −11→8
*T*_min_ = 0.931, *T*_max_ = 1.000	*k* = −27→20
9878 measured reflections	*l* = −9→10

### Refinement

**Table:**

Refinement on *F*^2^	Primary atom site location: structure-invariant direct methods
Least-squares matrix: full	Secondary atom site location: difference Fourier map
*R*[*F*^2^ > 2σ(*F*^2^)] = 0.033	Hydrogen site location: inferred from neighbouring sites
*wR*(*F*^2^) = 0.091	H-atom parameters constrained
*S* = 1.05	*w* = 1/[σ^2^(*F*_o_^2^) + (0.0529*P*)^2^ + 0.2963*P*] where *P* = (*F*_o_^2^ + 2*F*_c_^2^)/3
3020 reflections	(Δ/σ)_max_ < 0.001
226 parameters	Δρ_max_ = 0.17 e Å^−^^3^
0 restraints	Δρ_min_ = −0.19 e Å^−^^3^

### Special details

**Table:**

Geometry. All e.s.d.'s (except the e.s.d. in the dihedral angle between two l.s. planes) are estimated using the full covariance matrix. The cell e.s.d.'s are taken into account individually in the estimation of e.s.d.'s in distances, angles and torsion angles; correlations between e.s.d.'s in cell parameters are only used when they are defined by crystal symmetry. An approximate (isotropic) treatment of cell e.s.d.'s is used for estimating e.s.d.'s involving l.s. planes.
Refinement. Refinement of *F*^2^ against ALL reflections. The weighted *R*-factor *wR* and goodness of fit *S* are based on *F*^2^, conventional *R*-factors *R* are based on *F*, with *F* set to zero for negative *F*^2^. The threshold expression of *F*^2^ > σ(*F*^2^) is used only for calculating *R*-factors(gt) *etc*. and is not relevant to the choice of reflections for refinement. *R*-factors based on *F*^2^ are statistically about twice as large as those based on *F*, and *R*- factors based on ALL data will be even larger.

### Fractional atomic coordinates and isotropic or equivalent isotropic displacement parameters (Å^2^)

**Table:**

	*x*	*y*	*z*	*U*_iso_*/*U*_eq_	
N2	0.87262 (11)	0.11061 (4)	0.09736 (12)	0.0241 (2)	
N7	0.66539 (11)	0.16648 (4)	0.09585 (12)	0.0245 (2)	
H7N	0.7317	0.1969	0.1069	0.029*	
N14	0.06413 (11)	0.21713 (4)	0.08789 (13)	0.0261 (2)	
H14N	0.0015	0.1862	0.0829	0.031*	
N16	−0.13109 (11)	0.27154 (4)	0.11442 (12)	0.0244 (2)	
C1	0.73812 (12)	0.11242 (5)	0.11994 (13)	0.0210 (2)	
C3	0.95160 (13)	0.05927 (5)	0.12410 (15)	0.0280 (3)	
H3	1.0460	0.0578	0.1073	0.034*	
C4	0.90549 (14)	0.00852 (5)	0.17415 (16)	0.0298 (3)	
H4	0.9659	−0.0267	0.1916	0.036*	
C5	0.76666 (14)	0.01096 (5)	0.19811 (14)	0.0266 (2)	
H5	0.7314	−0.0229	0.2341	0.032*	
C6	0.68089 (13)	0.06259 (5)	0.16949 (14)	0.0234 (2)	
H6	0.5847	0.0646	0.1829	0.028*	
C8	0.51516 (13)	0.17821 (4)	0.09628 (13)	0.0207 (2)	
C9	0.37373 (13)	0.14622 (4)	−0.00512 (13)	0.0213 (2)	
H9	0.3791	0.1146	−0.0719	0.026*	
C10	0.22525 (13)	0.16003 (4)	−0.00954 (13)	0.0209 (2)	
H10	0.1301	0.1378	−0.0795	0.025*	
C11	0.21415 (13)	0.20611 (4)	0.08742 (13)	0.0212 (2)	
C12	0.35601 (13)	0.23827 (5)	0.18907 (14)	0.0237 (2)	
H12	0.3507	0.2698	0.2561	0.028*	
C13	0.50413 (13)	0.22456 (5)	0.19288 (13)	0.0225 (2)	
H13	0.5992	0.2470	0.2620	0.027*	
C15	−0.00317 (13)	0.27123 (5)	0.08107 (13)	0.0218 (2)	
C17	−0.20195 (13)	0.32340 (5)	0.10952 (15)	0.0273 (3)	
H17	−0.2923	0.3238	0.1333	0.033*	
C18	−0.15272 (14)	0.37602 (5)	0.07244 (15)	0.0271 (2)	
H18	−0.2051	0.4117	0.0736	0.032*	
C19	−0.02310 (14)	0.37485 (5)	0.03317 (14)	0.0260 (2)	
H19	0.0130	0.4100	0.0041	0.031*	
C20	0.05239 (13)	0.32251 (5)	0.03668 (14)	0.0247 (2)	
H20	0.1405	0.3210	0.0096	0.030*	
C21	0.49781 (18)	0.09257 (6)	0.65351 (19)	0.0418 (3)	
H21	0.5113	0.1219	0.7348	0.050*	
C22	0.41095 (17)	0.10534 (6)	0.4784 (2)	0.0407 (3)	
H22	0.3659	0.1433	0.4418	0.049*	
C23	0.39118 (14)	0.06342 (6)	0.36099 (16)	0.0338 (3)	
H23	0.3327	0.0726	0.2430	0.041*	
C24	0.45620 (13)	0.00641 (5)	0.41152 (14)	0.0260 (2)	
C25	0.43734 (15)	−0.03839 (6)	0.29296 (16)	0.0347 (3)	
H25	0.3784	−0.0304	0.1742	0.042*	

### Atomic displacement parameters (Å^2^)

**Table:**

	*U*^11^	*U*^22^	*U*^33^	*U*^12^	*U*^13^	*U*^23^
N2	0.0202 (4)	0.0227 (5)	0.0307 (5)	−0.0003 (3)	0.0131 (4)	−0.0019 (4)
N7	0.0202 (4)	0.0190 (4)	0.0381 (5)	−0.0005 (3)	0.0169 (4)	0.0010 (4)
N14	0.0216 (5)	0.0195 (4)	0.0415 (6)	−0.0003 (3)	0.0184 (4)	−0.0002 (4)
N16	0.0201 (4)	0.0214 (5)	0.0329 (5)	0.0005 (3)	0.0135 (4)	−0.0004 (4)
C1	0.0183 (5)	0.0210 (5)	0.0223 (5)	−0.0006 (4)	0.0084 (4)	−0.0027 (4)
C3	0.0220 (5)	0.0275 (6)	0.0365 (6)	0.0019 (4)	0.0154 (5)	−0.0039 (5)
C4	0.0278 (6)	0.0227 (6)	0.0369 (6)	0.0056 (4)	0.0135 (5)	−0.0008 (5)
C5	0.0275 (6)	0.0209 (5)	0.0287 (6)	−0.0019 (4)	0.0110 (5)	0.0001 (4)
C6	0.0208 (5)	0.0238 (5)	0.0266 (5)	−0.0013 (4)	0.0118 (4)	−0.0010 (4)
C8	0.0188 (5)	0.0189 (5)	0.0249 (5)	0.0023 (4)	0.0108 (4)	0.0042 (4)
C9	0.0236 (5)	0.0183 (5)	0.0236 (5)	0.0005 (4)	0.0123 (4)	−0.0003 (4)
C10	0.0191 (5)	0.0193 (5)	0.0227 (5)	−0.0016 (4)	0.0085 (4)	0.0015 (4)
C11	0.0197 (5)	0.0192 (5)	0.0258 (5)	0.0024 (4)	0.0115 (4)	0.0034 (4)
C12	0.0239 (5)	0.0211 (5)	0.0269 (5)	0.0013 (4)	0.0124 (4)	−0.0028 (4)
C13	0.0192 (5)	0.0209 (5)	0.0247 (5)	−0.0020 (4)	0.0081 (4)	−0.0011 (4)
C15	0.0177 (5)	0.0221 (5)	0.0236 (5)	0.0003 (4)	0.0078 (4)	−0.0012 (4)
C17	0.0216 (5)	0.0258 (6)	0.0364 (6)	0.0022 (4)	0.0151 (5)	−0.0016 (5)
C18	0.0248 (5)	0.0212 (5)	0.0313 (6)	0.0047 (4)	0.0098 (5)	−0.0005 (4)
C19	0.0250 (5)	0.0224 (5)	0.0255 (5)	−0.0019 (4)	0.0077 (4)	0.0019 (4)
C20	0.0213 (5)	0.0261 (5)	0.0276 (5)	−0.0002 (4)	0.0122 (4)	0.0015 (4)
C21	0.0534 (8)	0.0369 (7)	0.0546 (8)	−0.0155 (6)	0.0416 (7)	−0.0104 (6)
C22	0.0383 (7)	0.0321 (6)	0.0674 (9)	0.0032 (5)	0.0378 (7)	0.0111 (6)
C23	0.0219 (5)	0.0436 (7)	0.0356 (6)	0.0009 (5)	0.0132 (5)	0.0154 (5)
C24	0.0186 (5)	0.0352 (6)	0.0250 (6)	−0.0054 (4)	0.0108 (4)	0.0041 (4)
C25	0.0350 (6)	0.0458 (7)	0.0272 (6)	−0.0154 (5)	0.0178 (5)	−0.0032 (5)

### Geometric parameters (Å, °)

**Table:**

N2—C3	1.3415 (14)	C11—C12	1.3996 (15)
N2—C1	1.3466 (14)	C12—C13	1.3864 (15)
N7—C1	1.3732 (13)	C12—H12	0.9500
N7—C8	1.4129 (13)	C13—H13	0.9500
N7—H7N	0.9001	C15—C20	1.4066 (15)
N14—C15	1.3711 (14)	C20—C19	1.3763 (16)
N14—C11	1.4081 (13)	C20—H20	0.9500
N14—H14N	0.8999	C19—C18	1.3934 (16)
N16—C17	1.3436 (14)	C19—H19	0.9500
N16—C15	1.3441 (14)	C18—C17	1.3778 (16)
C1—C6	1.4087 (15)	C18—H18	0.9500
C3—C4	1.3778 (17)	C17—H17	0.9500
C3—H3	0.9500	C21—C25^i^	1.359 (2)
C4—C5	1.3938 (16)	C21—C22	1.405 (2)
C4—H4	0.9500	C21—H21	0.9500
C5—C6	1.3756 (15)	C22—C23	1.363 (2)
C5—H5	0.9500	C22—H22	0.9500
C6—H6	0.9500	C23—C24	1.4155 (18)
C8—C9	1.3947 (15)	C23—H23	0.9500
C8—C13	1.3955 (15)	C24—C25	1.4188 (18)
C9—C10	1.3877 (15)	C24—C24^i^	1.420 (2)
C9—H9	0.9500	C25—C21^i^	1.359 (2)
C10—C11	1.3934 (15)	C25—H25	0.9500
C10—H10	0.9500		
			
C3—N2—C1	117.53 (9)	C13—C12—H12	119.7
C1—N7—C8	125.32 (9)	C11—C12—H12	119.7
C1—N7—H7N	114.8	C12—C13—C8	120.73 (10)
C8—N7—H7N	118.1	C12—C13—H13	119.6
C15—N14—C11	125.87 (9)	C8—C13—H13	119.6
C15—N14—H14N	116.0	N16—C15—N14	114.89 (9)
C11—N14—H14N	117.9	N16—C15—C20	121.99 (10)
C17—N16—C15	117.47 (9)	N14—C15—C20	123.08 (10)
N2—C1—N7	114.91 (9)	C19—C20—C15	118.86 (10)
N2—C1—C6	121.85 (9)	C19—C20—H20	120.6
N7—C1—C6	123.22 (9)	C15—C20—H20	120.6
N2—C3—C4	124.62 (10)	C20—C19—C18	119.67 (10)
N2—C3—H3	117.7	C20—C19—H19	120.2
C4—C3—H3	117.7	C18—C19—H19	120.2
C3—C4—C5	117.38 (10)	C17—C18—C19	117.42 (10)
C3—C4—H4	121.3	C17—C18—H18	121.3
C5—C4—H4	121.3	C19—C18—H18	121.3
C6—C5—C4	119.73 (10)	N16—C17—C18	124.53 (10)
C6—C5—H5	120.1	N16—C17—H17	117.7
C4—C5—H5	120.1	C18—C17—H17	117.7
C5—C6—C1	118.88 (10)	C25^i^—C21—C22	120.32 (13)
C5—C6—H6	120.6	C25^i^—C21—H21	119.8
C1—C6—H6	120.6	C22—C21—H21	119.8
C9—C8—C13	118.59 (9)	C23—C22—C21	120.17 (12)
C9—C8—N7	121.38 (9)	C23—C22—H22	119.9
C13—C8—N7	119.95 (9)	C21—C22—H22	119.9
C10—C9—C8	120.75 (10)	C22—C23—C24	121.24 (12)
C10—C9—H9	119.6	C22—C23—H23	119.4
C8—C9—H9	119.6	C24—C23—H23	119.4
C9—C10—C11	120.75 (10)	C23—C24—C25	122.80 (11)
C9—C10—H10	119.6	C23—C24—C24^i^	118.50 (14)
C11—C10—H10	119.6	C25—C24—C24^i^	118.70 (14)
C10—C11—C12	118.52 (9)	C21^i^—C25—C24	121.07 (12)
C10—C11—N14	119.57 (9)	C21^i^—C25—H25	119.5
C12—C11—N14	121.83 (10)	C24—C25—H25	119.5
C13—C12—C11	120.67 (10)		
			
C3—N2—C1—N7	178.10 (10)	N14—C11—C12—C13	176.65 (10)
C3—N2—C1—C6	−0.10 (15)	C11—C12—C13—C8	−0.39 (16)
C8—N7—C1—N2	173.65 (10)	C9—C8—C13—C12	0.44 (16)
C8—N7—C1—C6	−8.18 (17)	N7—C8—C13—C12	177.21 (10)
C1—N2—C3—C4	−0.51 (17)	C17—N16—C15—N14	−179.99 (10)
N2—C3—C4—C5	0.15 (18)	C17—N16—C15—C20	−2.18 (16)
C3—C4—C5—C6	0.83 (17)	C11—N14—C15—N16	−168.34 (10)
C4—C5—C6—C1	−1.40 (16)	C11—N14—C15—C20	13.88 (17)
N2—C1—C6—C5	1.05 (16)	N16—C15—C20—C19	2.30 (16)
N7—C1—C6—C5	−176.99 (10)	N14—C15—C20—C19	179.93 (10)
C1—N7—C8—C9	−51.81 (15)	C15—C20—C19—C18	−0.34 (16)
C1—N7—C8—C13	131.51 (11)	C20—C19—C18—C17	−1.53 (16)
C13—C8—C9—C10	−0.20 (15)	C15—N16—C17—C18	0.14 (17)
N7—C8—C9—C10	−176.92 (9)	C19—C18—C17—N16	1.71 (18)
C8—C9—C10—C11	−0.10 (16)	C25^i^—C21—C22—C23	−0.01 (19)
C9—C10—C11—C12	0.16 (15)	C21—C22—C23—C24	0.29 (18)
C9—C10—C11—N14	−176.49 (9)	C22—C23—C24—C25	179.68 (11)
C15—N14—C11—C10	−138.39 (11)	C22—C23—C24—C24^i^	−0.23 (19)
C15—N14—C11—C12	45.08 (16)	C23—C24—C25—C21^i^	179.73 (11)
C10—C11—C12—C13	0.08 (16)	C24^i^—C24—C25—C21^i^	−0.36 (19)

Symmetry codes: (i) −*x*+1, −*y*, −*z*+1.

### Hydrogen-bond geometry (Å, °)

**Table:**

*D*—H···*A*	*D*—H	H···*A*	*D*···*A*	*D*—H···*A*
N7—H7N···N16^ii^	0.90	2.11	3.0027 (13)	175
N14—H14N···N2^iii^	0.90	2.13	3.0305 (13)	174

Symmetry codes: (ii) *x*+1, *y*, *z*; (iii) *x*−1, *y*, *z*.
